# Supplementary material for: A new species of Scapholeberis Schoedler, 1858 (Anomopoda: Daphniidae: Scapholeberinae) from the Colombian Amazon basin highlighted by DNA barcodes and morphology
Source: PeerJ. 2020 Sep 24;8:e9989. doi: 10.7717/peerj.9989 (PMC7520090; doi:10.7717/peerj.9989)
Supplement: Supplemental Information 2 — Clades suggested as species in coalescence tree [file peerj-08-9989-s002.pdf]

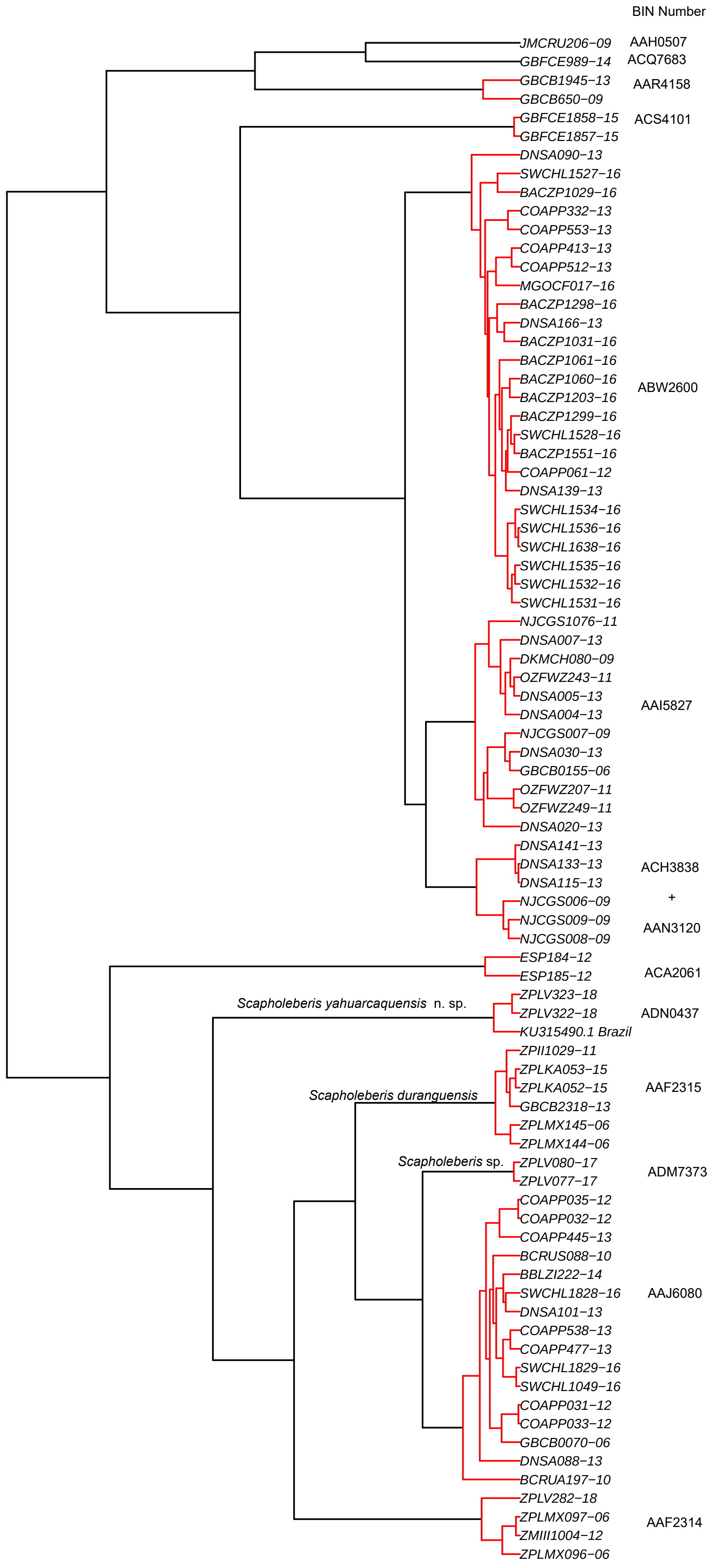

General mixed Yule coalescent (GMYC) model used as a species delimitation. In red after each branch is a species recognized by the model. Singletons are recognized as entities in this model.
